# Survival time of *Leptospira kirschneri* on strawberries

**DOI:** 10.1371/journal.pone.0237466

**Published:** 2020-08-13

**Authors:** Duygu Tekemen, Mathias Franz, Nadja S. Bier, Martin Richter, Karsten Nöckler, Enno Luge, Anne Mayer-Scholl

**Affiliations:** 1 Department of Biological Safety, German Federal Institute for Risk Assessment, Berlin, Germany; 2 Department Exposure, German Federal Institute for Risk Assessment, Berlin, Germany; University of California Riverside, UNITED STATES

## Abstract

In the past decade, two leptospirosis outbreaks occurred among strawberry harvesters in Germany, with 13, and 45 reported cases respectively. In both outbreaks, common voles (*Microtus arvalis*) infected with *Leptospira kischneri* serovar Grippotyphosa were identified as the most likely outbreak source. In an univariate analysis, eating unwashed strawberries was identified as one of the risk factors associated with *Leptospira* infection. The aim of this study was to evaluate the survival time of *L*. *kirschneri* serovar Grippotyphosa on strawberries under varying conditions. Strawberries were spiked with 5x10^9^ of both a laboratory reference strain (strain Moskva V) and an outbreak field strain (94-6/2007) of *L*. *kirschneri* serovar Grippotyphosa sequence type 110. Survival times were investigated in a fully crossed design with three incubation times (2h, 4h, 6h and 8h) and three temperatures (15°C, 21°C and 25°C) with three replicated for each condition. A wash protocol was developed and recovered *Leptospira* were determined by qPCR, dark field microscopy and culturing. Viable *L*. *kirschneri* of both the reference strain and the field strain were identified in all samples at 25°C and an incubation time of 2h, but only 1/9 (11%) and 4/9 (44%) of the samples incubated at 15°C were positive, respectively. Both reference and field strain were viable only in 2/9 (22%) at 25° after 6h. After an 8h incubation, viable *Leptospira* could not be identified on the surface of the strawberries or within the fruit for any of the tested conditions. Based on these results, the exposure risk of consumers to viable *Leptospira* spp. through the consumption of strawberries bought at the retail level is most likely very low. However, there is a potential risk of *Leptospira* infection by consumption of strawberries on pick-your-own farms.

## Introduction

Leptospirosis is a zoonotic disease, which occurs worldwide in both humans and animals [1, 2]. Pathogenic leptospires colonize the kidneys of animal hosts and are shed with the urine. Humans and animals are mostly infected through urine contaminated water or soil which come in contact with abrasions or cuts in the skin or mucous membranes [3]. The bacteria are able to cause a wide spectrum of variable clinical symptoms ranging from subclinical cases to life-threatening disease [4]. Depending on the infectious dose, the infecting serovar, the patient’s immune status and medical care given, case fatality rates in patients can reach > 50% [5].

Although the disease is considered rare in developed countries, low but persisting rates of autochthonous illness and case fatalities exist, with a suspected high underreporting rate [2, 6]. Historically, outbreaks of leptospirosis in Europe were associated with agricultural exposure [7]. In the past decades, recreational (e.g. water sports) and residential (e.g. gardening) related risk factors have increasingly gained importance and large outbreaks due to agricultural exposures have mostly disappeared. Yet recently, two agriculture-related leptospirosis outbreaks in 2007 and 2014 were documented among strawberry harvesters in Germany, with 13 and 45 reported cases, respectively [8, 9]. In both outbreaks, common voles (*Microtus arvalis*) infected with *Leptospira kischneri* serovar Grippotyphosa ST110 were identified as the most likely outbreak source. In an outbreak investigation, harvesting in the rain, contact with mud and rodents, the presence of hand lesions and consumption of unwashed strawberries were associated with an increased risk of infection [8].

The risk of infection after contact with any environmental source depends on the ability of *Leptospira* bacteria to survive and persist in the environment [10]. The described outbreak investigation led to the question if *Leptospira* spp. can survive long enough on strawberries to pose a public health risk. Therefore, the aim of this study was to evaluate the survival times of *L*. *kirschneri* serovar Grippotyphosa on strawberries under varying temperature conditions.

## Materials and methods

### Bacterial strains and cultivation

Both a laboratory (strain Moskva V) and a field strain (94-6/2007) of *L*. *kirschneri* serovar Grippotyphosa sequence type 110 were used in this study. The field strain 94-6/2007 was isolated during a leptospirosis outbreak among strawberry harvesters in 2007 from a common vole (*Microtus arvalis*) trapped on the strawberry fields of the affected strawberry farm. During dissection of the animal the entire kidney was collected for isolation of *Leptospira* spp. The kidney was minced using a scalpel, transferred to liquid Ellinghausen McCullough Johnson Harris medium (EMJH, Difco^TM^, Michigan, USA) supplemented with 0.1% 5-fluorouracil (5Fu, w/v) and 2% rabbit serum (v/v), incubated aerobically at 29 ± 1° C and examined under a dark-field microscope for the presence of *Leptospira* over a period of 4 weeks weeks. Strain identity was confirmed by *secY* sequencing [11] and multi locus sequence typing according to scheme 1 [12].

For the spiking experiments, the strains were grown in 100 ml EMJH medium at 29 ± 1°C for seven days to reach a concentration of approximately 10^9^ cells/ml. For harvesting, the cell suspensions were centrifuged at 10,000 x g for 40 minutes at room temperature and the cell pellets were resuspended in 1 ml of filtered rainwater (0.2 μm, cellulose acetate). Rainwater was chosen for bacterial dilution, as it is a natural water source. Cells were counted in a cell counting chamber (Thoma, depth 0.02 mm) and the suspensions were adjusted to 5x10^10^ cells/ml with rainwater.

### Preparation of strawberries for spiking

For each experiment, fresh strawberries (Fragaria x ananassa ‘Sonata’, grown in Rövershagen, Germany) were obtained from local retailers on farmers markets in Steglitz, Lankwitz and Wedding, Berlin. After elimination of the carpels using scissors, the strawberries were washed under flowing distilled water, treated with UV light for 30 minutes to eliminate other microorganisms and left to dry. During the drying period, strawberries were turned every 10 minutes.

### Spiking and incubation of *Leptospira* on strawberries

Each strawberry was spiked with a total of 5x10^9^ bacteria by pipetting 100 μl of the cell suspension (5x10^10^ cells/ml) on 10 different spots of the strawberry surface. Spiked strawberries were incubated at different incubation times (2h, 4h, 6h and 8h) at 15°C, 20°C or 25°C. The selected temperatures are based on the average minimum and maximum summer temperatures in Germany.

To guarantee a constant humidity during incubation, a small container with water was left in the incubator and the humidity was monitored constantly using a data logger (LOG32, ED, European Distribution Company, Grezzago, Italy). On each spiking day, 12 strawberries were spiked and incubated with one temperature: three strawberries were examined after two hours, three after four hours, three after six hours and the final three after eight hours. On the following days, the same procedure was repeated with further incubation temperatures.

To verify that the leptospires do not actively enter the strawberries, the fruits were spiked in triplicate (5x10^9^ cells), homogenized after an eight-hour incubation time according to protocol B and the resulting suspension was tested for viable *Leptospira* by culture (see below).

### Development of a protocol for the efficient removal of *Leptospira* from the strawberry surface

In order to verify that the spiked bacteria could be retrieved from the strawberries during the survival experiments, two protocols (A and B) were compared and evaluated.

Protocol A: Spiked strawberries were carefully transferred into 300 ml Erlenmeyer flasks with 100 ml freshly prepared EMJH medium supplemented with 0.1% 5Fu (w/v) and incubated on an orbital shaker at 100 rpm for 10 minutes to remove the bacteria from the strawberries.

Protocol B: The spiked strawberries were carefully transferred in Interscience filter-bags XR 400 (400 ml max. blending volume, 280 microns filter porosity), with 100 ml freshly prepared EMJH medium supplemented with 0.1% 5Fu (w/v) and homogenized using the BagMixer® 400 P (Interscience, Paris, France) for 1 minute with a speed of 7 strokes per second.

In the resulting suspensions of both protocols a maximum concentration of 5x10^7^ bacteria/ml was expected. For comparison of both protocols, the recovery rate was determined by quantifying the number of *Leptospira* using direct microscopy with a cell counting chamber and qPCR as endpoint.

To estimate the maximum possible recovery, 5x10^9^ bacteria were transferred in triplicates directly into the medium in either the stomacher bags or the Erlenmeyer flasks without any strawberries, respectively. These reference samples were examined directly after spiking by direct microscopy and qPCR. The recovery rate for each protocol was calculated as ratio between the number of detected *Leptospira* in the sample and the reference sample.

### Detection of *Leptospira* spp.

#### DNA extraction and qPCR

To estimate the recovery rate of the bacteria from protocol A or B, 1 ml of the resulting suspensions were centrifuged at 10,000 x g for 40 min and DNA was extracted from the pellet using the DNeasy Blood and KitQIAamp DNA Mini Kit (Qiagen, Hilden, Germany) according to the manufacturer’s instructions. For detection of leptospiral DNA, qPCR targeting a partial sequence (242 bp) of the *lipl32* gene was used, which encodes a 32-kDa leptospiral major outer membrane lipoprotein [13]. The qPCR reaction was set up using the PerfeCTa qPCR ToughMix UNG L-ROX Mastermix (QuantaBio, Beverly, USA), 0.5 μM of primer *lipL32*-45F (AAGCATTACCGCTTGTGGTG) [14] and *lipL32-286*Rb (GAACTCCCATTTCAGCGAT) [15] 0,1μM of a sequence-specific TaqMan probe (FAM-5´-AA AGC CAG GAC AAG CGC CG-3´-BHQ) [14] and 5 μL template DNA). Thermal cycling was carried out with the 7500 real-time PCR system (Applied Biosystems, USA). The following amplification protocol was performed: a pre-incubation step at 50°C for 2 min, initial denaturation at 95°C for 10 min, followed by 45 cycles of amplification at 95°C for 15 s and 60°C for 60 s. For bacterial quantification, a standard curve utilising a 10-fold serial dilution of leptospiral DNA (*L*. *interrogans* Serovar icterohaemorrhagiae strain RGA, 106–10^1^ genome equivalents/PCR) was generated by plotting the quantification cycles (Cq) of the serial dilution against the log of the template concentration. The corresponding regression line was used to determine the concentration of unknown samples from their Cq-values.

After spiking the strawberries with 5x10^9^ cells/ml, a maximum number of 4,2x10^6^ genome equivalents (GE) were expected in the qPCR reaction.

#### Quantification by dark field microscopy

Viable bacteria were counted using dark field microscopy and a cell counting chamber (Thoma, depth 0.02 mm). 20 small squares in a row were counted and the number of cells/μl was determined using the formula: counted cells x dilution factor x 1000μl/ depth of a cell (0.02 mm) x square of a cell (0.0025 mm^2^) x number of counted cells.

#### Detection of viable *Leptospira kirschneri* by dark field microscopy and culture

For both strains a qualitative assessment was performed where a trial was determined as positive, when a single motile *Leptospira* was detected in the washing solution by dark field microscopy. Quantification of motile bacteria was only assessed with the *Leptospira* reference strain. In this case, the median number of motile bacteria was calculated if more than one replicate sample per time point and temperature contained live bacteria. The ratio between the median number of viable bacteria in the sample and a reference (5x10^9^ *Leptospira* spiked in triplicate into the Erlenmeyer flasks without any strawberries and examined directly by direct microscopy) was determined.

Additionally, cultures were prepared for both strains by inoculating 4 ml EMJH medium supplemented with 0.1% 5Fu (w/v) with 0.4 ml of the recovered suspensions. Cultures were examined using dark field microscopy after 24 hours and thereafter every 7 days for 4 weeks to detect motile bacteria. After four weeks, most cultures were overgrown by accompanying bacterial flora and could not be further examined.

### Statistical analysis

To determine how variations in temperature, strain and incubation time affected the survival of the pathogens, a generalized linear mixed model with a binomial error distribution was used. Survival as a binary variable was included as response. As fixed effects the following predictors were included: temperature and incubation time as continuous variables and strain as a categorical variable. In addition, the trial number and the replicate number of each trial were included as nested random effects. The analysis was conducted in the statistical software R [16] using the package lme4 [17].

## Results

### Evaluation of bacterial recovery protocols A and B

**Fig 1:**
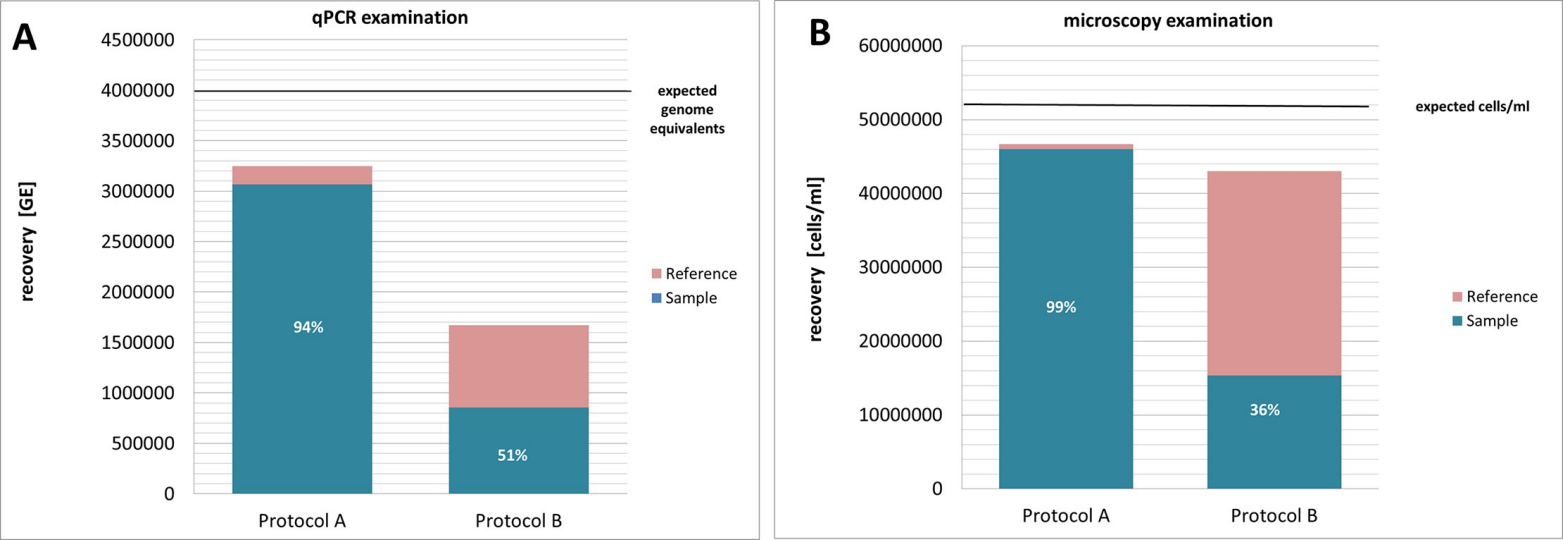
**Recovery of *Leptospira kirschneri* from strawberries using the washing protocol (protocol A) and the homogenization protocol (protocol B) after spiking 5x10**^**9**^ **leptospires on the strawberry surface.** Examination of bacterial recovery with qPCR (A) and by microscopy (B). The recovery rate was calculated as the ratio between the number of detected bacteria in the sample and the reference sample. The black line shows the expected number of genome equivalents 4,2x10^6^ genome equivalents (GE) (A) or 5x10^7^ bacteria/ml (B) in the final sample.

Washing the strawberries in flasks (protocol A) showed the highest recovery of 3.1x10^6^ GE/ml (94% compared to the reference sample) by quantification using qPCR and 4.6x10^7^ cells/ml (99%) using microscopy (Fig 1). In contrast, using the homogenization protocol B, a recovery of only 8.6x10^5^ GE/ml (51%) was estimated by qPCR and 1.6x10^7^ cells/ml (36%) by cell counting (Fig 1). In addition to the lower recovery rates, which could be due to inhibitory factors found in the homogenized fruits, the visual examination under the microscope was more challenging after homogenization due to strawberry residues in the samples.

Based on these results, protocol A was chosen for all further experiments in the present study.

### Quantification of viable *Leptospira kirschneri*

At 15°C only one replicate was countable after two hours incubation time, indicating a 4% survival (2x10^6^ bacteria/ml) in comparison to the reference sample (4.8x10^7^ bacteria/ml). In contrast, on average 8% (2.8x10^6^ bacteria/ml, 6/9 replicates positive) and on average 25% (1.2x10^7^ bacteria/ml, 9/9 replicates positive) of *Leptospira* survived at 21°C (reference sample 4.8x10^7^ cells/ml) and 25°C (reference sample 4.6x10^7^ cells/ml), respectively.

### Survival of *Leptospira kirschneri* on strawberries

**Fig 2:**
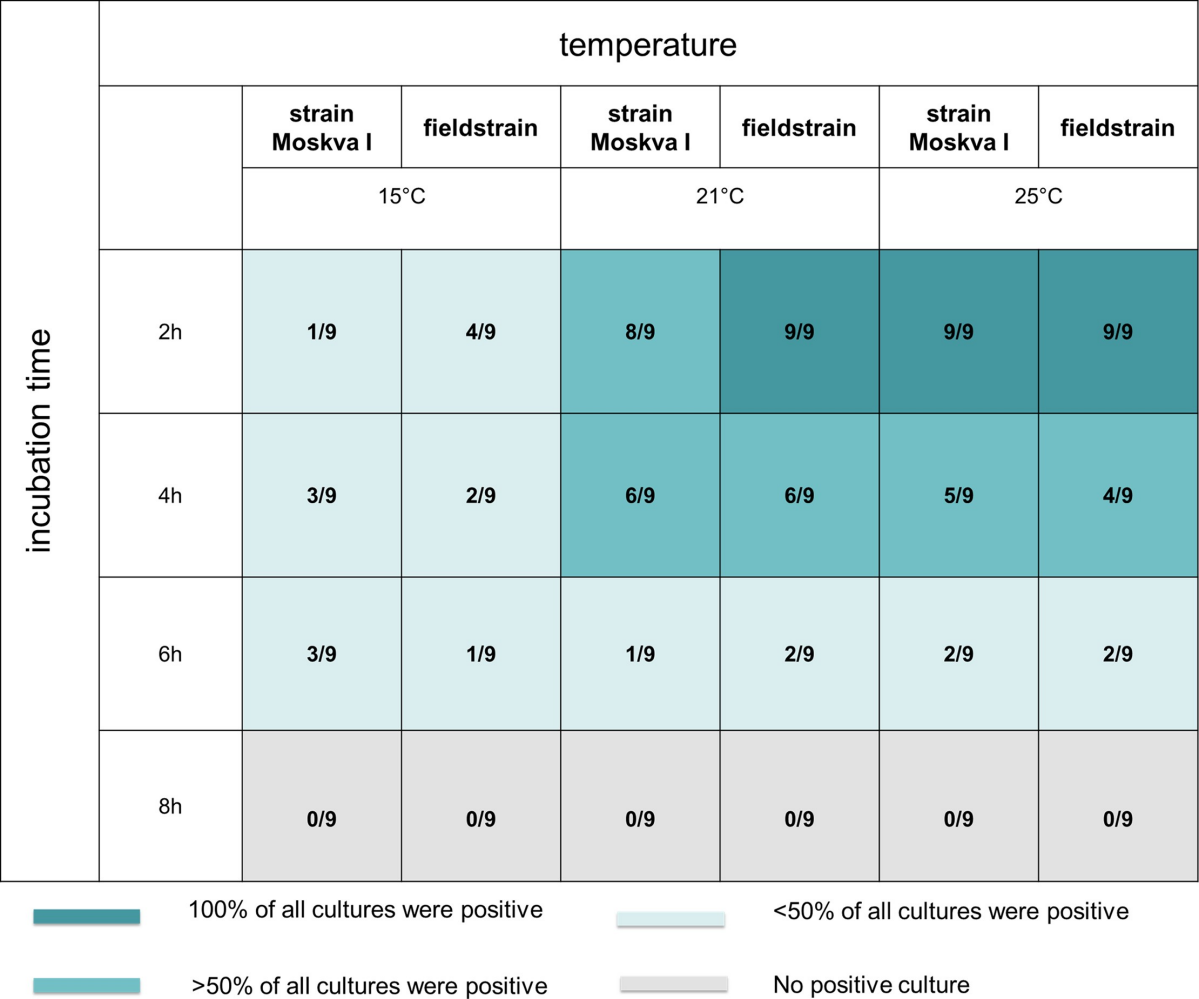
Survival of *Leptospira kirschneri* serovar Grippotyphosa strain Moskva V (reference strain) and the field strain (94-6/2007) after incubation on strawberries for different time-periods and at varying temperature examined by dark field microscopy of the cultures. Ratios in the cells depict the number of culture positive replicates per total number of replicates.

All replicates showing viable Leptospira during microscopy yielded positive cultures, but viable bacteria were only detected by direct microscopy in 22/35 (63%) of culture positive samples. Due to the higher sensitivity of the culture method, all statistical analyses were based on the results of this method. We did not find any indication for a statistically significant difference between both strains (z = 0, p = 1). However, the leptospiral survival significantly decreased with increasing incubation time (z = -7.01, p<0.001) and significantly increased with increasing temperature (z = 4.10, p<0.001) (Fig 2). The highest survival of 100% was observed for the conditions of 2h incubation time with 21 and 25°C for both strains. The lowest survival of 0% was observed for all conditions of 8h incubation time.

## Discussion

Currently, there is a very limited knowledge about the persistence of pathogenic leptospires in the environment [10]. Yet, the pathogen's ability to survive and remain infectious is critical to determine the risk of transmission to a host. The emergence of leptospirosis cases related to fieldwork on strawberry farms, and the fact that consumption of strawberries was considered a potential risk factor in an univariate regression analysis, prompted us to examine the survival of *Leptospira* on strawberries under controlled laboratory conditions.

Our findings indicate that the pathogenic strain *L*. *kirschneri* serovar Grippotyphosa cannot survive on strawberries for extended periods-of-time. Although bacterial survival increased with higher incubation temperatures, the pathogens only survived on the strawberries for a less than 8 hours, independent of temperature. The current study was performed under the assumption that strawberries dry during picking, packaging and transport. As described by Karaseva *et al*., [18] survival of *Leptospira* spp. in soil is positively correlated with the moisture of the soil [18]. This indicates that the reduction of moisture in any environment reduces leptospiral survival times and thus the risk of human infection.

Leptospiral survival studies have thus far only been performed for environmental matrices such as water, soil and mud. Once the pathogens are excreted into the environment, a multitude of factors such as pathogen species, temperature, pH-value, moisture, humidity, UV light, salt and mineral concentrations and the presence of other microorganisms affect survival [10, 19–21]. In soil, the reported survival times span from a few hours to 193 days [22–28]. In tap water, distilled water, sea- and river water different *Leptospira* species survived between a few hours and 20 months [21, 28–32]. In this study we could show that leptospiral survival significantly increased with increasing incubation temperatures. This corroborates the study from Fontaine *et al*., [32], who demonstrated that leptospiral survival is temperature dependent as the survival time increased from 130 days at 4°C to 263 days at 20°C and to 316 days at 30°C in fresh water [32].

The association of this disease with rain, temperature and extreme weather events is well established and mirrored in the frequency of leptospirosis outbreaks [10]. The majority of agricultural associated leptospirosis outbreaks in Central Europe were associated with temperatures > 18°C, heavy rains and water puddles on the fields [8, 9, 33–35].

Leptospirosis remains a multifactorial disease where reservoir animal abundance (mostly rodents), *Leptospira* prevalence in the reservoir host and human behaviour (e.g. use of personal protective equipment, covering of lesions) have a marked effect on human exposure risk [36]. Further, the transmission from host to host depends largely on the survival time of the pathogens in the environment [28, 31].

Typically, *Leptospira* infection occurs when the bacteria penetrate the body through mucosal membranes or skin cuts and enter the bloodstream of the host. Yet, an oral intake of pathogens through e.g. swallowing water is thought to be an important route of entry [37]. A very limited number of studies have implicated oral transmission of *Leptospira* [38, 39], and there are currently no published studies that used the oral or intranasal inoculation route to establish infection [10]. To our knowledge this is one of the first studies looking at *Leptospira* spp. as a possible food contaminate.

Under the assumption, that strawberries are usually not eaten directly from the field but are transported to retail level for a certain time period under dry conditions, the results obtained here indicate that the consumption of strawberries marketed at retail or market level poses a very low risk of human infection with *Leptospira kischneri*. The risk could be further reduced by washing the fruits prior to consumption to facilitate the removal of any bacteria from the surface.

The question remains if strawberries consumed directly during harvest, by either field workers or consumers on pick-your-own fields carry a higher risk. Here, leptospiral survival on strawberries is most probably sufficiently long to pose a risk of human infection, as shown in our study. The effect of increased moisture and varying temperatures, either due to wetter and more humid weather conditions or transport conditions, on the survival of the pathogen on strawberries remains to be verified.
